# Development of a Novel Nomogram to Predict Major Adverse Cardiac Events in Patients with Chronic Total Occlusion

**DOI:** 10.7150/ijms.94644

**Published:** 2024-04-22

**Authors:** Wenjie Chen, Jinghua Liu, Yuchen Shi

**Affiliations:** Center for Coronary Artery Disease (CCAD), Beijing Anzhen Hospital, Capital Medical University, Beijing Institute of Heart, Lung and Blood Vessel Diseases, Beijing 100029, China.

**Keywords:** Coronary artery disease, Nomogram, Myocardial Metabolism Imaging, Positron-Emission Tomography

## Abstract

**Objectives:** To create a nomogram using single photon emission computed tomography (SPECT) myocardial perfusion imaging and ^18^F-FDG positron emissions tomography (PET) gated myocardial metabolism imaging to forecast major adverse cardiovascular events (MACE) in chronic total occlusion (CTO) patients treated with optimal medical therapy (OMT).

**Methods:** A total of 257 patients who received OMT between January 2016 and December 2021 were included in this retrospective study. Patients were randomly divided into development (n=179) and validation (n=78) cohorts. A thorough evaluation was conducted, encompassing clinical features and imaging analysis, which involved assessing myocardial perfusion and metabolism. Independent risk factors were identified using least absolute shrinkage and selection operator (LASSO) and multivariate Cox regression analyses. Calibration curves and decision curve analysis (DCA) were used to evaluate the clinical usefulness.

**Results:** In the development cohort, 53 patients (29.6%) experienced MACE out of 179 patients, while in the validation cohort, MACE occurred in 23 (29.5%) patients out of 78. The PET-left ventricular end-systolic volume (P-ESV) (HR 1.01; 95% CI 1.003-1.017; p=0.003), hibernating myocardium / total perfusion defect (HM/TPD) (HR 1.053; 95% CI 1.038-1.069; p<0.001), PET-left ventricular ejection fraction (P-LVEF) (HR 0.862; 95% CI 0.788-0.943; p=0.001), and left anterior descending branch (LAD) (HR 2.303; 95% CI 1.086-4.884; p=0.03) were significantly associated with MACE and were used to develop the nomogram. The nomogram demonstrated excellent discrimination with C-indexes of 0.931 and 0.911 in the development and validation cohorts. DCA determined that the model exhibited a considerably superior net advantage in predicting MACE.

**Conclusion:** A new nomogram integrating clinical factors and imaging features was created to predict the risk of MACE in patients with CTO.

## 1. Introduction

Chronic total occlusion (CTO) may result in serious consequences such as angina, myocardial infarction, arrhythmias, and heart failure. Early detection and treatment are crucial in order to mitigate these risks 1. While percutaneous coronary intervention (PCI) may be a viable treatment option for CTO 2, the therapeutic advantages of revascularization versus optimal medical therapy (OMT) remain controversial 3. Current guidelines recommend revascularization for CTO patients with myocardial perfusion deficits exceeding the 10% threshold, indicating a favorable prognosis 4. However, due to the intricacies of CTO lesions and the challenges associated with revascularization, some patients with compromised general health or a history of surgical failures often opt for OMT 5.

Regarding patient management, in addition to traditional clinical characteristics and blood markers, multiple imaging parameters can assist in risk stratification 6. Myocardial perfusion imaging (MPI), owing to its ability to assess cardiac perfusion status, has been extensively validated in CTO patients for diagnostic categorization, therapeutic responsiveness assessment, and prognostication outcomes 7-9. While myocardial metabolism imaging is well-recognized for its favorable assessment of cardiac viability 10, its application in CTO patients has been limited 11,12. The integration of medical information and imaging measurements may yield a better model for guiding treatment decisions in CTO patients.

To address this, we established a novel nomogram based on single photon emission computed tomography (SPECT) MPI and ^18^F-FDG positron emissions tomography (PET) gated myocardial metabolism imaging to predict the prognosis of CTO patients undergoing OMT during follow-up.

## 2. Methods

### 2.1. Patient Selection

**Figure 1:**
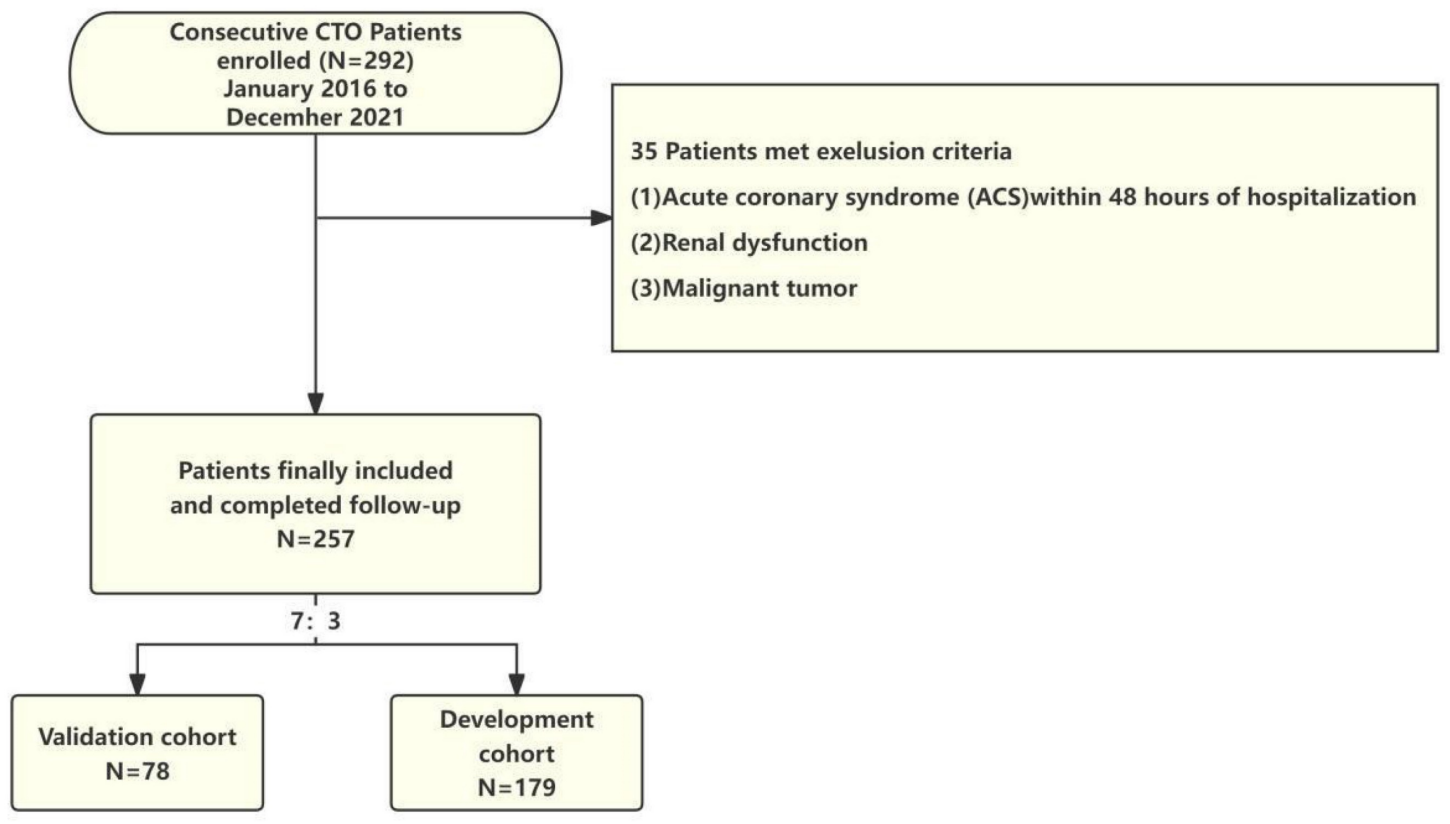
Flow chart for patient selection.

Patients with CTO who received OMT due to failed revascularization or high surgical risk from January 2016 to December 2021 were selected from the case registry at Beijing AnZhen Hospital. In this study, the CTO criteria is "Definite CTO." CTO was defined, in accordance with established standards, as a complete occlusion (TIMI 0 flow) lasting at least 3 months in a major coronary artery with a vessel diameter of at least 2.5 mm, as confirmed through coronary angiography (CAG) 2. OMT refers to the utilization of optimal medical therapy to manage patients with CTO. This therapy includes the administration of antiplatelet agents, lipid-lowering medications, and cardiovascular protective drugs such as beta-blockers, ACE inhibitors, or ARBs, aiming to alleviate symptoms and improve cardiac function 3. The inclusion criteria included: (1) CTO lesion location: proximal and mid left anterior descending branch (LAD); proximal left circumflex artery (LCX) or large obtuse marginal branches; proximal, mid, or distal dominant right coronary artery (RCA), (2) Completion of MPI during hospitalization. Exclusion criteria included (1) acute coronary syndrome (ACS) within 48 hours of hospitalization, (2) presence of kidney dysfunction, and (3) malignant tumor. Upon enrollment, all patients were randomly assigned to either the development cohort or the validation cohort at a ratio of 7:3. Figure 1 presents the flowchart for patient selection. The study protocol received approval from the Human Research Ethics Committee of Beijing Anzhen Hospital, Capital Medical University, Beijing, China, approved the study protocol (Approval No.2022177X), and adhered to the ethical guidelines outlined in the Declaration of Helsinki (revised in 2013). As the study was retrospective, patient consent was not required.

### 2.2. Image Acquisition and Analysis

Methods for acquiring and processing MPI images were consistent with previous descriptions 13. The Siemens Biograph mCT PET/CT system (Siemens AG, Germany) performed gated myocardial metabolism imaging with ^18^F-FDG PET/CT, following the American Heart Association guideline 14. As detailed earlier 15, the patients underwent an overnight fasting period, followed by oral glucose administration and intravenous insulin injection to regulate blood sugar levels. Image acquisition lasted 10 minutes, capturing 8 frames per cardiac cycle, with a matrix size of 128 x 12 and a zoom factor of 1.0.CT data (120 kV, 11 mAs) was utilized for attenuation correction. The examinations utilized an X5-1 probe operating at a 1.0-5.0 MHz frequency range. Tomographic images of the left ventricle in the short-axis, vertical, and horizontal long-axis orientations were obtained by combining MPI images reconstructed from 4 iterations and 8 subsets with ^18^F-FDG PET-gated myocardial metabolism images of 2 iterations and 21 subsets 16. The Cedars-Sinai QPS software from the United States was employed for measurements, including total perfusion defect (TPD) percentage, hibernating myocardium (HM) percentage, HM/TPD, and the extent of SCAR percentage. The evaluation of HM and infarcted myocardium in the left ventricle involved a combination of visual and semi-quantitative analysis methods, following previously described protocols 16. Quantitative gated SPECT (QGS) software (Cedars-Sinai, USA) was used to assess left ventricular ejection fraction (LVEF), end systolic volume (ESV), end diastolic volume (EDV).

### 2.3. Data collection and follow-up

The analysis included the examination of various factors such as demographic characteristics (age, gender), comorbidities (hypertension, diabetes, hyperlipidemia), MPI (P-EDV, P-ESV, P-LVEF, HM/TPD, TPD, extent of SCAR), coronary angiography (CTO lesion location and extent), and the medications prescribed upon discharge (Dual antiplatelet therapy (DAPT), Statins, Beta-blocker, SGLT-2 inhibitors). An MPI assessment was conducted upon admission. A standardized questionnaire was used to assess clinical endpoints during telephonic interviews every 6 months. During the follow-up period, this study aimed to determine the frequency of MACE, encompassing cardiac death; readmission due to worsening heart failure (WHF), re-revascularization (revascularization performed again three months after discharge). The occurrence of acute myocardial infarction (MI) was also considered. The duration between the MPI study and the occurrence of MACE was calculated to determine the time to the MACE, and mortality status was independently verified using official death certificates.

### 2.4. Statistical analysis

The effective sample size in prediction nomogram (development and validation) was determined by the number of outcome events. It was defined to have at least 10 outcome events per variable (EPV) to ensure accuracy 17. Based on previous large-scale RCT study, the incidence of MACE during a three-year follow-up period is around 24.9% 18. In comparison to prior literature, the population included in our study is expected to have a MACE incidence rate of 29.5%. Four variables were selected through LASSO regression, and in order to allow for four or fewer predictor factors in the final multivariable cox regression model, we estimate that we would need 133 or more patients. Our sample size and the number of outcome events exceed the EPV method, thus potentially providing reliable estimates. The normality of quantitative data was assessed using the Shapiro-Wilk test. Quantitative data that followed a normal distribution were presented as x̄±s, and the independent samples t-test was used for between-group comparisons. Quantitative data that did not follow a normal distribution were presented as M (Q₁, Q₃), and the Mann-Whitney U test was used for between-group comparisons. Qualitative data were presented as percentages (%), and between-group comparisons were conducted using the chi-square test or Fisher's exact test. The LASSO was then utilized to identify prognostic elements linked to MACE. The chosen features, with non-zero coefficients in the LASSO regression analysis, indicating their potential as survival predictors were included in the multivariable Cox regression analysis constructed through stepwise regression analysis. Hazard ratios (HR) and 95% confidence intervals (CI) were calculated to quantify the extent of risk. For the variables with a significance level of P<0.05, incorporate them into the multivariate Cox regression model to construct a nomogram. Utilizing the nomogram involved drawing lines representing predictors upwards to acquire points, which were then summed and placed on the 'Total Points' axis. The model's predictive accuracy and conformity were assessed using the Receiver Operating Characteristic (ROC) and calibration curves. Bootstrap analysis with 1000 repeated samplings was performed and a Time-area under the ROC curve (AUC) relationship was plotted to enhance the robustness of the results and compensate for the small sample size. DCA was utilized to evaluate the model's overall advantage for patients. Patient survival was illustrated using Kaplan-Meier (KM) plots, with distinctions assessed through the log-rank examination. Finally, we deployed a website for predicting MACE risk in CTO patients online. Statistical significance was determined if the two-sided P-value was below 0.05. The statistical analyses were conducted using SPSS 24.0 from IBM in the USA and R software version 4.3.2.

## 3. Results

### 3.1. Baseline characteristics

For the final analysis, 257 patients (with an average age of 62.0 (56.0;68.0) years were included (Table 1). The majority of participants were males (90.7%, n=257), with an average LVEF of 20.0 (15.0;26.0)%. Continuous monitoring of all individuals was maintained until the occurrence of MACE, with a median follow-up duration of 24.0 (19.0, 30.0) months. Following the follow-up, 76 patients, accounting for 29.6% of the participants, achieved the combined MACE endpoint. The development cohort included 179 cases, and the validation cohort comprised 78 cases. A comparison of their characteristics revealed no significant disparities between the two cohorts in clinical parameters and imaging indexes (P > 0.05).

### 3.2. Feature selection and construction of nomogram

**Figure 2:**
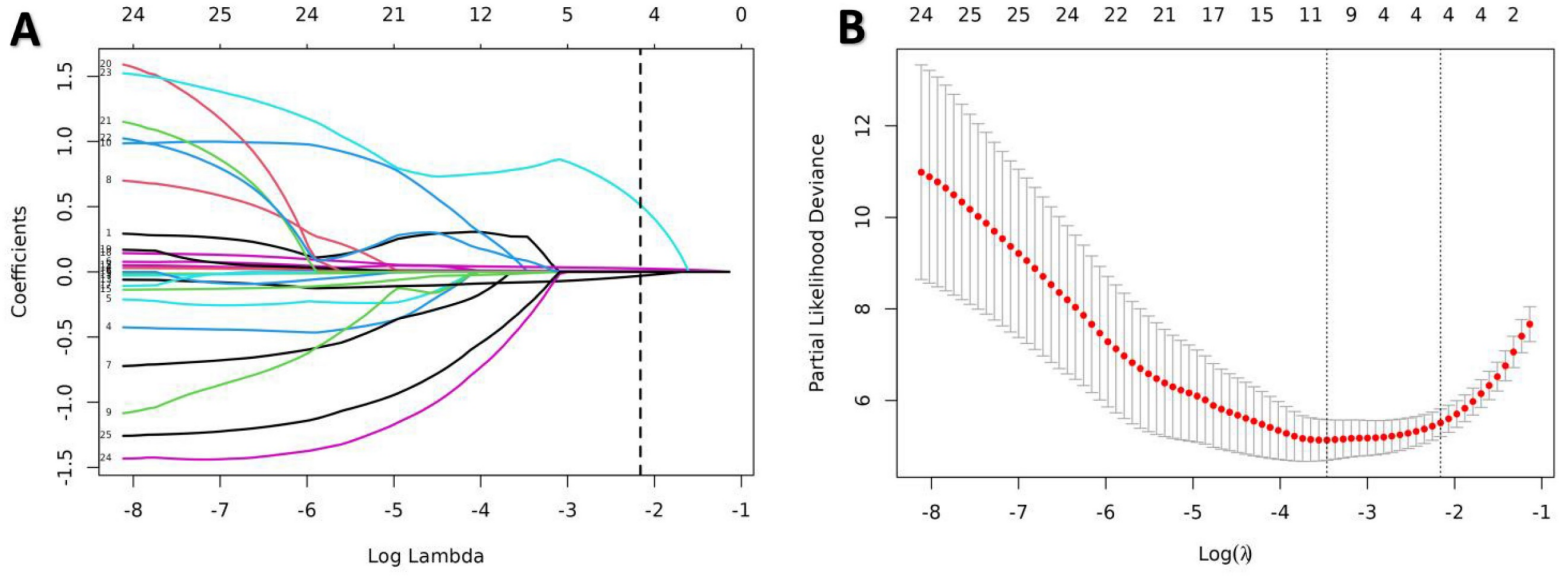
Predictors selection using the LASSO Cox regression model. **(A)** Optimal parameter (lambda) selection in the LASSO model used tenfold cross-validation via minimum criteria. **(B)** LASSO coefficient profiles of all the variables. A coefficient profile plot was produced against the log(λ) sequence. A dotted vertical line was drawn at the value selected using tenfold cross-validation, where optimal lambda (with the 1-SE criteria) resulted in candidate predictive variables. LASSO, least absolute shrinkage, and selection operator.

**Figure 3:**
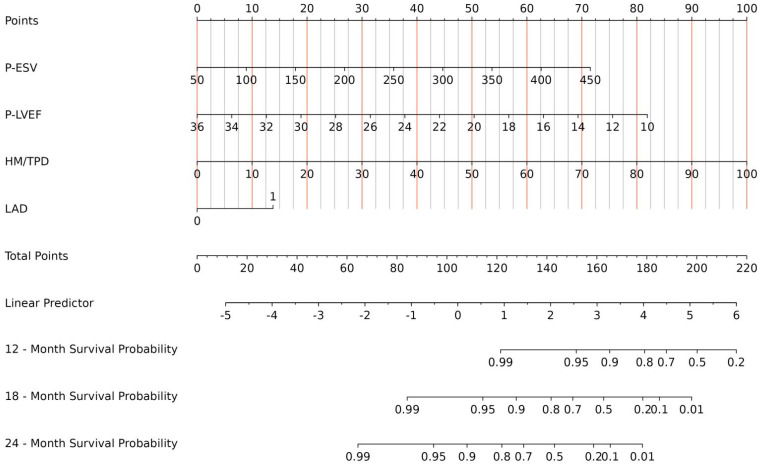
A nomogram prediction model to predict the MACE of CTO.

All indicators in Table 1 were included in the Lasso regression analysis. Through 10-fold cross-validation, the optimal λ value was selected, and the results showed that the Lasso regression obtained a model with excellent performance at Log(λ) = 4. Subsequently, the variables with non-zero coefficients were identified as: P-ESV (coefficient: 0.00699), HM/TPD (coefficient: 0.0300), P-LVEF (coefficient: -0.0567), LAD (coefficient: 0.3532) (Fig. 2). These four variables were then included in the multivariate Cox regression through stepwise regression analysis. The results indicated that P-ESV (HR 1.01; 95% CI 1.003-1.017; p=0.003), HM/TPD (HR 1.053; 95% CI 1.038-1.069; p<0.001), P-LVEF (HR 0.862; 95% CI 0.788-0.943; p=0.001), and LAD CTO (HR 2.303; 95% CI 1.086-4.884; p=0.03) were independent risk factors for MACE occurrence in CTO patients. Refer to Table 2 for details. A multivariate Cox regression model was established to incorporate these significant risk factors. The risk of MACE occurrence was indicated by plotting the total scores on the prediction axis, assigned to each of the four separate predictors: P-ESV, HM/TPD, P-LVEF, and LAD CTO (Fig. 3). A representative example would be a patient with a LAD lesion (12 points), P-LVEF of 20% (50 points), HM/TPD of 50 (50 points) and P-ESV of 35 (55 points). According to the nomogram, the patient's cumulative score amounted to 167, corresponding to roughly 0.86, 0.4, and 0.08 probabilities of survival at 12-, 18-, and 24-month, respectively.

### 3.3. Verification of the nomogram

**Figure 4:**
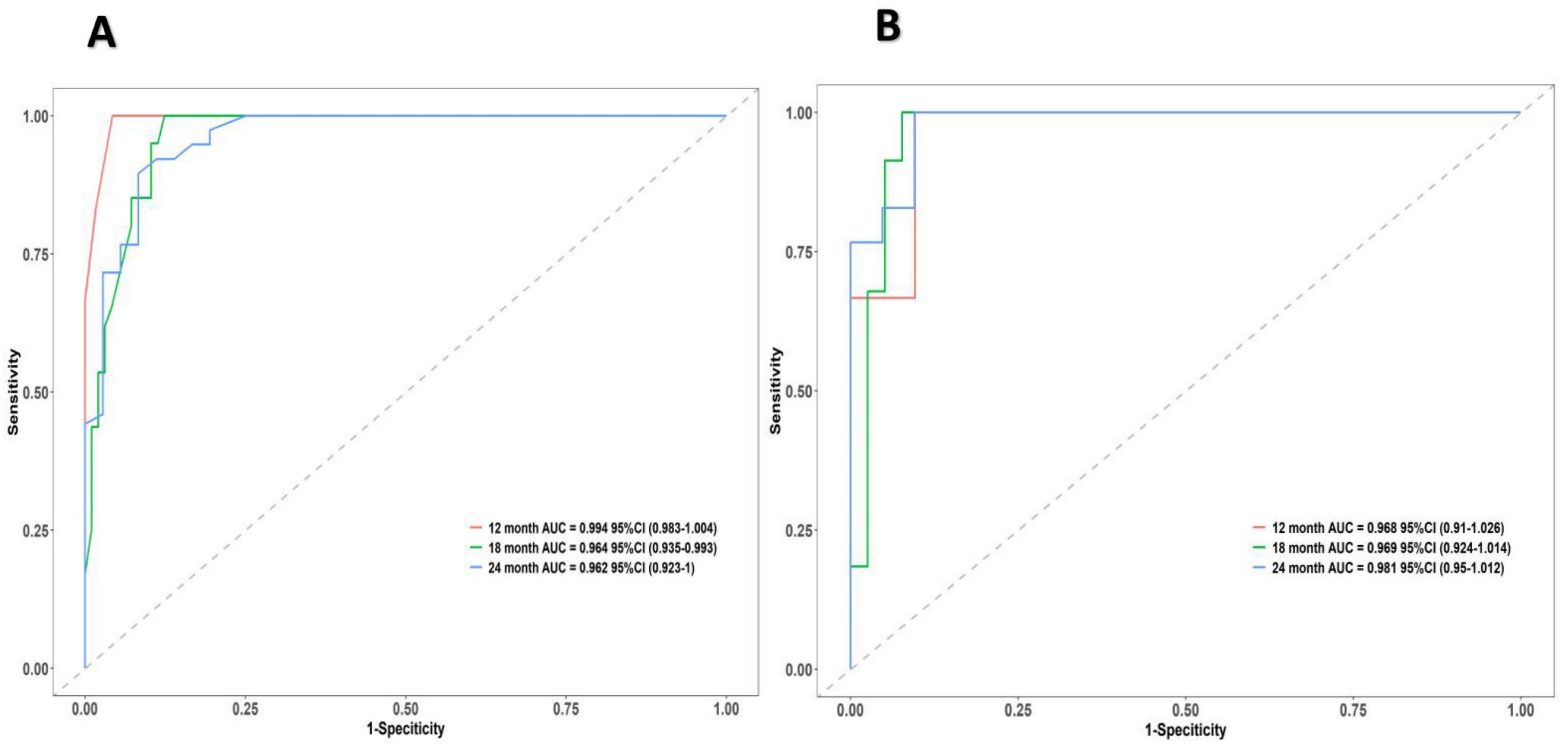
ROC curves of the nomogram. ROC for Development group, and Validation group, receiver operating characteristic; AUC, area under the ROC curve.

**Figure 5:**
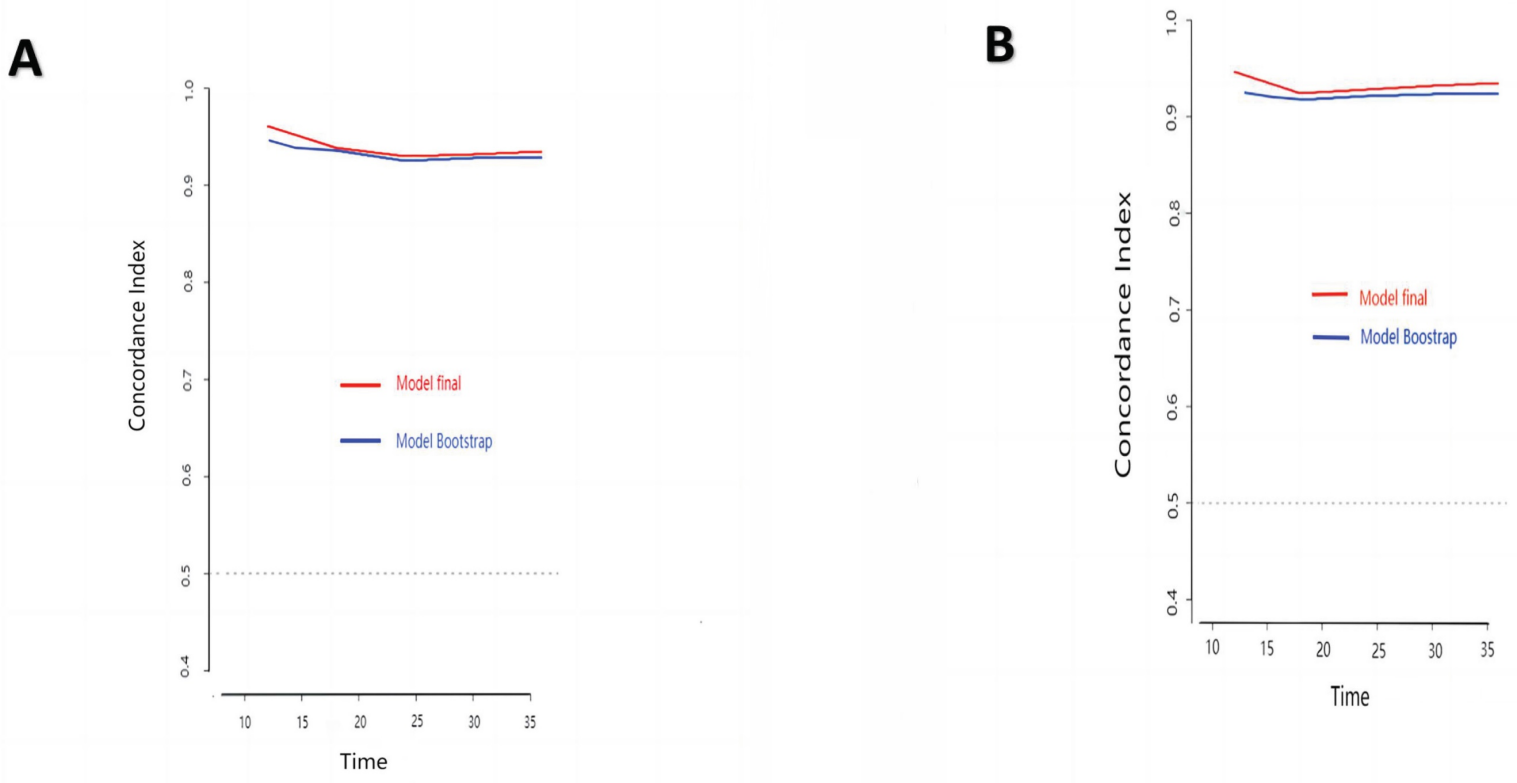
Time-AUC based on Bootstrap analysis for Development group, and Validation group.

**Figure 6:**
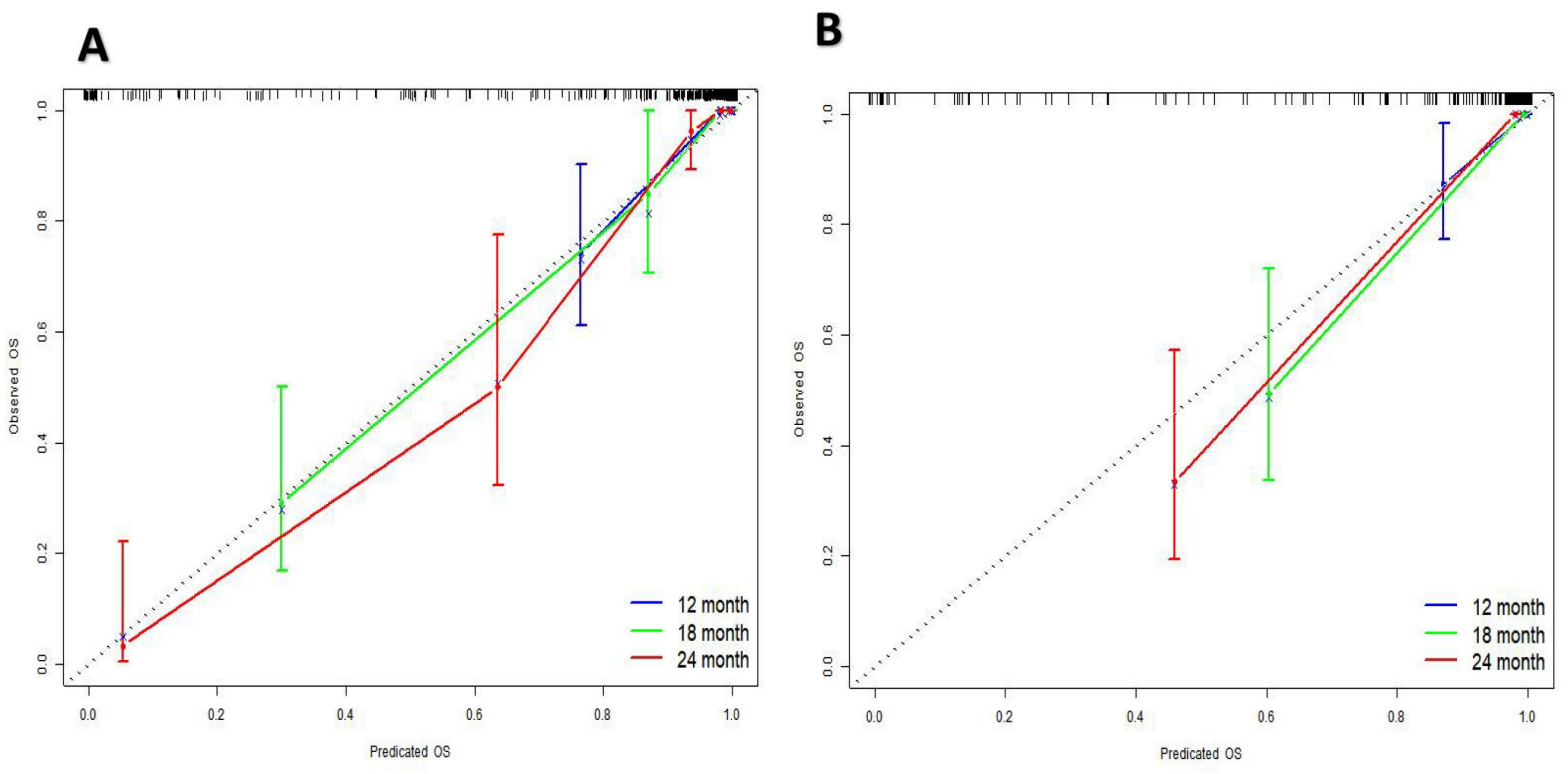
Calibration curves for Development group, and Validation group.

**Figure 7:**
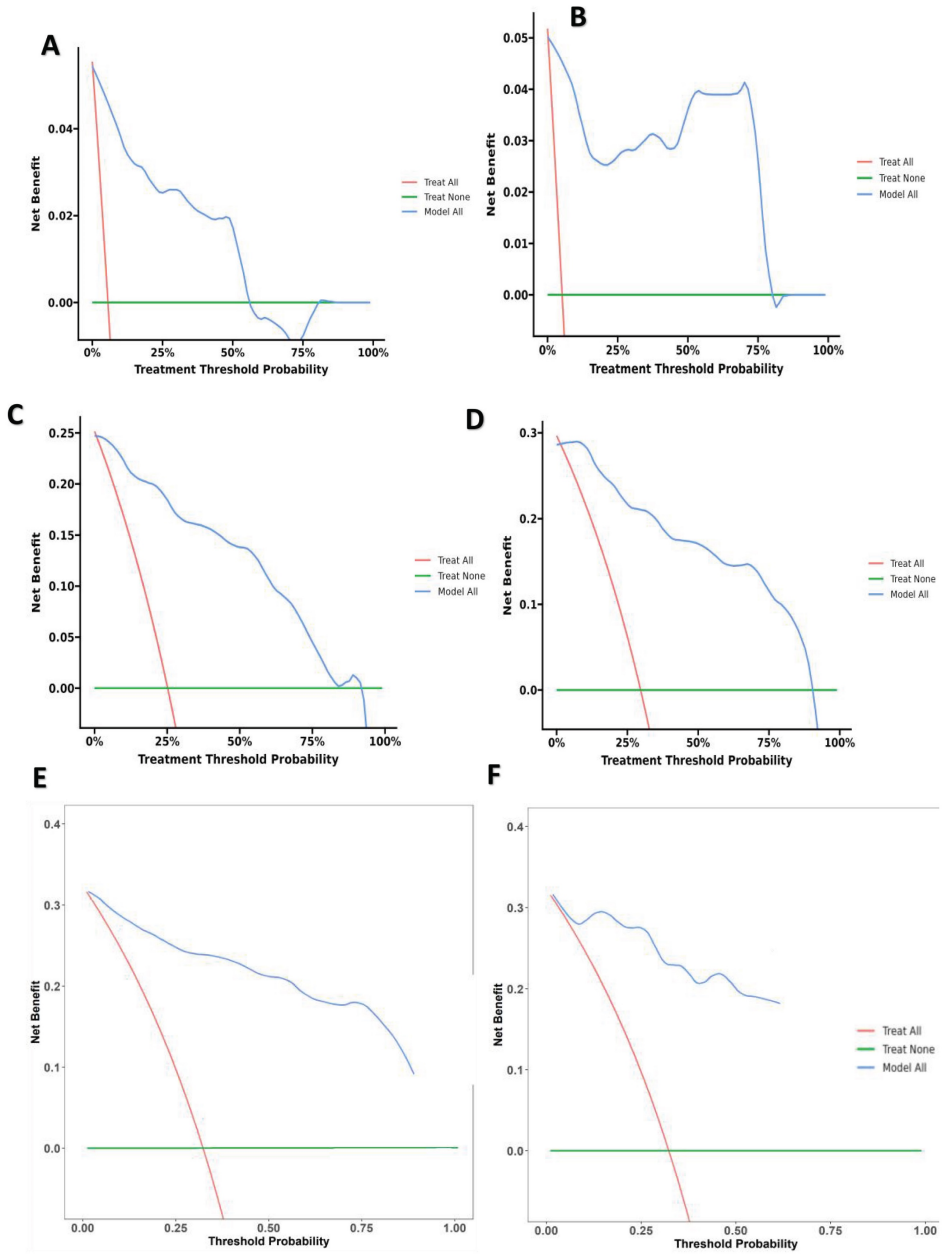
** (A)** 12-month Decision curve analysis for Development group, **(B)** 12-month Decision curve analysis for Validation group, **(C)** 18-month Decision curve analysis for Development group, **(D)** 18-month Decision curve analysis for Validation group, and E 24-month Decision curve analysis for Development group, 24-month Decision curve analysis for Validation group.

**Figure 8:**
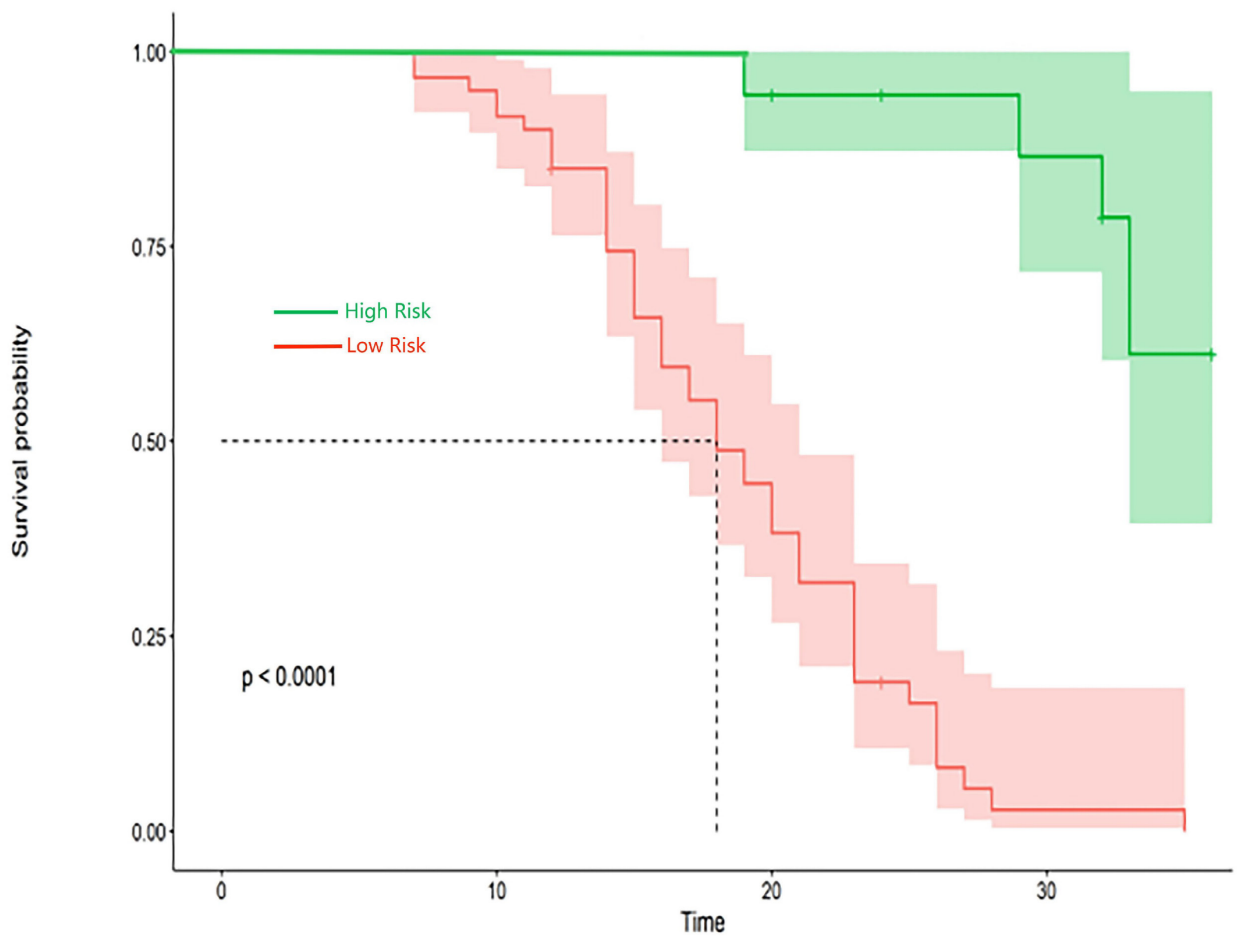
Kaplan-Meier survival curves in patients with CTO.

**Figure 9:**
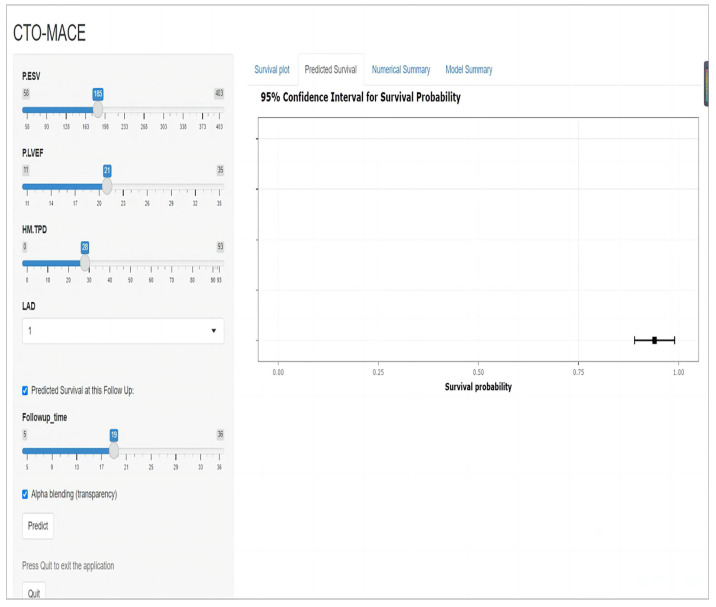
A website for predicting MACE risk in CTO patients online (https://cto-mace.shinyapps.io/dynnomapp/).

The research assessed the predictive model's discriminatory capacity and calibration to verify the nomogram's effectiveness. The model demonstrated a high discriminative ability, with the C-index of 0.942, 95% CI (0.918-0.966) and 0.943, 95% CI (0.908-0.978) for the development and validation cohorts, respectively. The ROC curves demonstrated the AUC values for forecasting the likelihood of survival at 12-, 18- and 24-month, measuring 0.994 ,0.964, and 0.962 in the development group, and 0.968, 0.969 and 0.981 in the validation group, respectively (Fig. 4). Bootstrap analysis with 1000 repeated samplings was used to compensate for the small sample size and Time-AUC relationships were plotted for the development and validation sets (Fig. 5). These results reinforce the model's outstanding discriminatory ability. Additionally, calibration plots revealed a strong concordance between the predicted survival probability and the observed outcomes, as the model closely followed the 45-degree diagonal line (Fig. 6). This suggests that the model is well-calibrated. Furthermore, in this study, DCA curves for individual variables were separately plotted and compared with the nomogram. The results of DCA showcased that the utilization of the nomogram for estimating survival rates at 12-, 18-, and 24-month enhanced the clinical risk prediction across various threshold probabilities (Fig. 7). We summed the scores of each column line chart variable to obtain the total risk score for each patient. All patients were divided into high-risk and low-risk groups based on the critical value. The Kaplan-Meier curve results show that the difference in survival rates between high-risk and low-risk patients in the entire cohort is statistically significant. The nomogram demonstrated exceptional discriminative ability, as evidenced by a Log-Rank p-value of less than 0.01 (Fig.8). Finally, we deployed a website for predicting MACE risk in CTO patients online (https://cto-mace.shinyapps.io/dynnomapp/) (Fig. 9).

## 4. Discussion

The clinical course of CTO displays a spectrum of differences, encompassing individuals with mild symptoms, ventricular arrhythmias, severe heart failure, and sudden cardiac death 19, 20. Recognizing the diverse manifestations of CTO is necessary, since a singular clinical indicator falls short in providing a comprehensive risk assessment for affected patients. Therefore, there is a critical need to develop a tailored and precise strategy that combines various clinical indicators. Such an approach aims to improve predictive accuracy and cost-efficiency, addressing the growing demands for individuals diagnosed with CTO.

The pivotal pathophysiological transformations within CTO encompass myocardial stunning and hibernation 21. Prolonged reduction in resting blood flow leads to chronic ventricular wall motion abnormalities, initiating myocardial hibernation and subsequently contributing to HF and ischemic cardiomyopathy 22. While myocardial metabolism imaging is the gold standard for identifying viable myocardium 10, the controversy surrounding its use in treating patients with CTO persists. Randomized controlled revascularization trials have yet to conclusively demonstrate the definitive advantages of viability testing.

The substudy of PARR-2 has provided evidence for identifying stunning and hibernation myocardium using PET to assess the extent of mismatch between perfusion and metabolism. Following revascularization, patients with a more significant mismatch, indicative of a higher hibernating myocardium, showed enhanced clinical outcomes 23. In contrast, the results from the REVIVED-BCIS2 investigation did not reveal any advantages in detecting viable myocardium 18. In response to this, the introduction of a new parameter, HM/TPD, has been proposed in this study. HM/TPD displayed a stronger correlation with MACE compared to HM alone. This study emphasizes the importance of viability assessments for patients with CTO to enhance risk evaluation. Indeed, if the blood supply to HM is not timely revascularized, relying solely on pharmaceutical treatment may prove insufficient, increasing the risk of deteriorating cardiac function and increased mortality.

Impaired left ventricular dilation and systole function are integral factors in the progression of the disease. The prognostic significance of LVEF in heart conditions is well-established, and various imaging methods can be employed to assess it 24. Despite the conventional use of echocardiography in assessing ventricular function, it is still plagued by drawbacks, such as low reproducibility and limited temporal resolution 25. Mounting evidence supports MPI as a promising tool with clinical significance for measuring function. Gated myocardial PET, compared to echocardiography, offers greater accuracy in assessing cardiac function 26. Therefore, in this investigation, the prognosis was independently predicted by LVEF measured through gated myocardial PET rather than echocardiography. Individuals with impaired left ventricular function exhibited an increased incidence of cardiovascular death and advanced HF.

Moreover, studies suggest that the increase in the end-systolic volume of the left ventricle could be linked to the decline in heart function and the progression of heart conditions. In individuals with coronary artery disease, restricted blood flow to the coronary leads to myocardial damage and ventricular remodeling, ultimately increasing ESV 27. An increased ESV may suggest inadequate diastolic function, wherein the heart fails to relax and fill properly, thereby decreasing its pumping efficiency. White et al. 28 mentioned in their research involving 605 patients who experienced post-acute myocardial infarction that ESV was identified as the primary risk factor for mortality using Cox multivariate regression analysis. The results of this current research are consistent with previous findings, confirming ESV's independent and combined predictive value in patients with CTO.

Additionally, our research highlights the historical emphasis on the importance of CTO location as a predictive factor, impacting the outcomes of revascularization procedures. Among the current cohort, individuals with CTO lesions in the LAD tended towards more unfavorable outcomes. This tendency is typically attributed to the extensive supply range of the LAD, which triggers sympathetic nervous system activation when its dominant territory experiences ischemia. On the other hand, blockage of the RCA usually triggers the stimulation of the vagal nerve, thus playing a defensive function in preventing dangerous heart rhythm abnormalities. Therefore, optimal results are expected from revascularizing CTO lesions in the LAD, followed by LCX. Conversely, the least significant improvements are linked to the revascularization of the RCA. When confronted with multi-branch disease and multiple CTO lesions, strategic prioritization should favor the LAD and the LCX 29, 30.

Using LASSO regression and multivariate Cox regression analysis, our study has developed a predictive scoring chart that combines clinical data and imaging characteristics. The selection of LASSO regression analysis over univariate COX regression analysis addressed issues such as multicollinearity among myocardial perfusion and metabolism imaging parameters and guards against potential model overfitting. However, since LASSO achieves variable selection by penalizing coefficients, this may lead to overestimation or underestimation of certain variables, resulting in the model ignoring or underestimating these variables. Therefore, we use cross-validation to evaluate model performance and stability, reducing selection bias. Multivariate Cox regression is sensitive to unobserved risk factors. If important risk factors are not included in the model, it can lead to omitted variable bias. Therefore, after selecting variables through LASSO, before entering into multivariate Cox regression analysis, we not only consider whether the variables are statistically significant but also focus on their clinical significance. Only after considering both aspects comprehensively, these variables are included in the Cox regression to reduce omitted variable bias.

Previous predictive models have incorporated various traditional clinical indicators, including general clinical characteristics and laboratory parameters, to assess the severity and prognosis of CTO patients 31, 32. Their application in clinical practice remains challenging, because some models omit crucial MPI variables. As the clinical benefits of PCI and OMT are still unclear, we encourage the performance of viability myocardial testing in patients with a confirmed diagnosis of CTO. After MPI, relevant parameters are obtained. The nomogram is used to identify high-risk patients, especially those with a significant amount of viable myocardium and LAD-CTO, to determine whether PCI should be performed on the patient. Utilizing this nomogram may reduce unnecessary PCI for some patients, thus alleviating the burden on patients. However, due to the relatively high cost of MPI, this could potentially be a barrier to the clinical implementation of the nomogram.

## 5. Limitations

Regarding the limitations of this study, we recognize that this was a single-center retrospective study, introducing the potential for selection bias. While cross-validation and repeated sampling methods were used to mitigate the impact of small sample size, there may still be a potential reduction in statistical power due to the small sample size. Further validation is needed to assess the applicability of this nomogram to diverse populations, particularly considering geographic and ethnic variations. Despite being a major cardiovascular center in China, our institution can gather patients from across the country. However, for other ethnicities, extensive multicenter and multinational studies with large sample sizes are required for further validation. Despite the aforementioned limitations, this prediction model is intended to aid clinicians in evaluating the prognosis of CTO patients, facilitating the development of tailored treatment strategies.

## 6. Conclusions

In conclusion, the HM/TPD, assessed through MPI and myocardial metabolism imaging, has been identified as a novel and independent predictor of MACE in CTO patients. A nomogram incorporating HM/TPD offers prognostic value, enhancing risk stratification for these patients.

## Figures and Tables

**Table 1:** The baseline clinical characteristics of both the Development and Validation cohort.

	[ALL] N=257	Validation cohort N=78	Development cohort N=179	P
MACE:				1
NO	181 (70.4%)	55 (70.5%)	126 (70.4%)	
YES	76 (29.6%)	23 (29.5%)	53 (29.6%)	
Clinical demographic				
Gender, n (%)				0.571
male	233 (90.7%)	69 (88.5%)	164 (91.6%)	
female	24 (9.34%)	9 (11.5%)	15 (8.38%)	
Age (years) M (Q₁, Q₃)	62.0 [56.0;68.0]	62.0 [55.0;68.0]	61.0 [57.0;68.0]	0.684
Base Heart Rate(bpm), M (Q₁, Q₃)	72.0 [68.0;80.0]	72.0 [65.0;80.0]	72.0 [68.0;80.0]	0.742
Diabetes mellitus, n (%)				1
NO	171 (66.5%)	52 (66.7%)	119 (66.5%)	
YES	86 (33.5%)	26 (33.3%)	60 (33.5%)	
Dyslipidemia, n (%)				0.975
NO	110 (42.8%)	34 (43.6%)	76 (42.5%)	
YES	147 (57.2%)	44 (56.4%)	103 (57.5%)	
Hypertension, n (%)				1
NO	116 (45.1%)	35 (44.9%)	81 (45.3%)	
YES	141 (54.9%)	43 (55.1%)	98 (54.7%)	
Previous MI, n (%)				0.534
NO	167 (65.0%)	48 (61.5%)	119 (66.5%)	
YES	90 (35.0%)	30 (38.5%)	60 (33.5%)	
Previous PCI, n (%)				0.412
NO	140 (54.5%)	46 (59.0%)	94 (52.5%)	
YES	117 (45.5%)	32 (41.0%)	85 (47.5%)	
Current tobacco use, n (%)				0.276
NO	69 (26.8%)	25 (32.1%)	44 (24.6%)	
YES	188 (73.2%)	53 (67.9%)	135 (75.4%)	
Alcohol consumption, n (%)				0.771
NO	199 (77.4%)	59 (75.6%)	140 (78.2%)	
YES	58 (22.6%)	19 (24.4%)	39 (21.8%)	
MPI parameters				
P-EDV (mL/m2) M (Q₁, Q₃)	225 [167;276]	227 [162;292]	223 [167;258]	0.685
P-ESV (mL/m2) M (Q₁, Q₃)	173 [116;231]	173 [119;250]	173 [116;223]	0.648
P-LVEF (%) M (Q₁, Q₃)	20.0 [15.0;26.0]	19.5 [15.0;25.0]	20.0 [15.0;26.0]	0.412
PBW M (Q₁, Q₃)	138 [102;168]	144 [120;172]	132 [102;168]	0.142
PSD M (Q₁, Q₃)	36.1 [28.4;47.5]	37.8 [31.2;49.1]	35.0 [27.9;45.0]	0.111
HM (%) M (Q₁, Q₃)	10.0 [4.00;20.0]	9.00 [4.25;19.5]	10.0 [4.00;19.0]	0.951
TPD (%) M (Q₁, Q₃)	45.0 [34.0;54.0]	43.0 [34.0;53.8]	46.0 [34.0;54.0]	0.779
HM/TPD M (Q₁, Q₃)	25.0 [9.00;44.0]	18.0 [9.25;40.0]	25.0 [8.50;44.0]	0.977
Scar (%) M (Q₁, Q₃)	30.0 [19.0;42.0]	30.0 [19.0;42.8]	30.0 [19.0;42.0]	0.977
Baseline medications				
ARNI:				0.65
NO	59 (23.0%)	16 (20.5%)	43 (24.0%)	
YES	198 (77.0%)	62 (79.5%)	136 (76.0%)	
Statin: YES	257 (100%)	78 (100%)	179 (100%)	.
DAPT: YES	257 (100%)	78 (100%)	179 (100%)	.
Bblocker: YES	257 (100%)	78 (100%)	179 (100%)	.
CCB:				0.829
NO	195 (75.9%)	58 (74.4%)	137 (76.5%)	
YES	62 (24.1%)	20 (25.6%)	42 (23.5%)	
Angiographic characteristics				
CAD-1v:				0.458
NO	178 (69.3%)	51 (65.4%)	127 (70.9%)	
YES	79 (30.7%)	27 (34.6%)	52 (29.1%)	
CAD-2v:				0.529
NO	139 (54.1%)	45 (57.7%)	94 (52.5%)	
YES	118 (45.9%)	33 (42.3%)	85 (47.5%)	
CAD-3V:				1
NO	198 (77.0%)	60 (76.9%)	138 (77.1%)	
YES	59 (23.0%)	18 (23.1%)	41 (22.9%)	
LAD-CTO:				1
NO	182 (70.8%)	55 (70.5%)	127 (70.9%)	
YES	75 (29.2%)	23 (29.5%)	52 (29.1%)	
RCA-CTO:				0.689
NO	135 (52.5%)	39 (50.0%)	96 (53.6%)	
YES	122 (47.5%)	39 (50.0%)	83 (46.4%)	
LCX-CTO:				0.764
NO	143 (55.6%)	45 (57.7%)	98 (54.7%)	
YES	114 (44.4%)	33 (42.3%)	81 (45.3%)	

MI = Myocardial Infarction; LVEF = Left Ventricular Ejection Fraction; P-EDV = PET Left Ventricular End-Diastolic Volume, P-ESV = PET Left Ventricular End-Systolic Volume; TPD = Total Perfusion Defect; HM = Hibernating Myocardium; PSD = Phase Standard Deviation; PBW= Phase Histogram Bandwidth; ACEI = Angiotensin-Converting Enzyme Inhibitor; ARB = Angiotensin Receptor Blocker; ARNI = Angiotensin Receptor-Neprilysin Inhibitor; RCA = Right Coronary Artery; LAD = Left Anterior Descending Branch; LCX = Left Circumflex Artery

**Table 2:** Multivariable Cox Analysis for MACE in CTO Patients.

Characteristics	B	SE	HR	CI	Z	P
P-ESV	0.01	0.003	1.01	1.003-1.017	2.983	0.003
P-LVEF	-0.148	0.046	0.862	0.788-0.943	-3.232	0.001
HM/TPD	0.052	0.007	1.053	1.038-1.069	6.937	0
LAD	0.834	0.383	2.303	1.086-4.884	2.176	0.03
